# Supplementary material for: Open-loop analog programmable electrochemical memory array
Source: Nat Commun. 2023 Oct 4;14:6184. doi: 10.1038/s41467-023-41958-4 (PMC10550916; doi:10.1038/s41467-023-41958-4)
Supplement: Supplementary file 1 — Supplementary Information [file 41467_2023_41958_MOESM1_ESM.pdf]

Supplementary Information

Open-Loop Analog Programmable Electrochemical Memory Array

Peng Chen, Fenghao Liu, Peng Lin*, Peihong Li, Yu Xiao, Bihua Zhang and Gang Pan*

*Correspondence to penglin@zju.edu.cn, gpan@zju.edu.cn

This PDF files includes:

Supplementary Figure 1. Materials characterization of pristine YSZ/WOx interface.

Supplementary Figure 2. Transfer characteristics of ECRAMs.

Supplementary Figure 3. Pulse width modulation of ECRAMs.

Supplementary Figure 4. Asymmetry-nonlinearity factor calculation.

Supplementary Figure 5. Conductance modulation of an ECRAM over wide dynamic range.

Supplementary Figure 6. Cycling performance of ECRAM device.

Supplementary Figure 7. Endurance test of the device.

Supplementary Figure 8. Schematic diagrams of the read operations in an array.

Supplementary Figure 9. Write errors of continuous pattern programming.

Supplementary Figure 10. Write errors of pattern programming with 1~3 cycles.

Supplementary Figure 11. Fabrication process of an ECRAM array.

Supplementary Figure 12. Experimental set-up for array operation.

Supplementary Figure 13. Evaluation of crosstalk disturbance in half-selected ECRAM devices.

Supplementary Figure 14. Simulation of half-selected disturbance in larger arrays.

Supplementary Table 1. Summary of input parameters for in-situ training tasks.

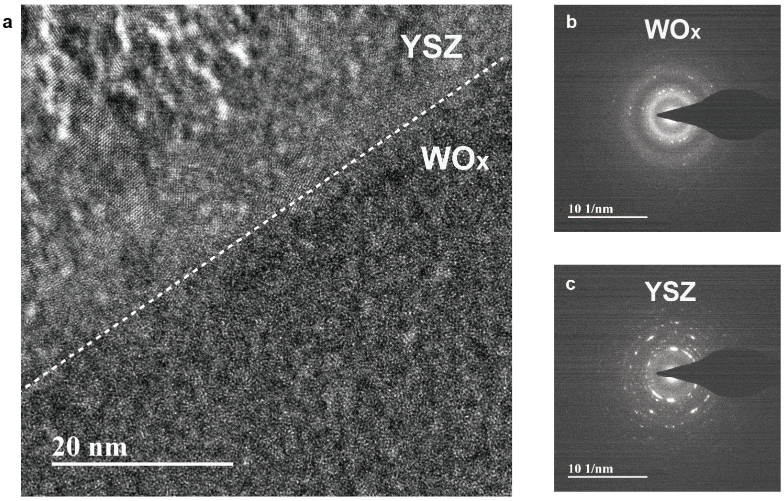

Supplementary Figure 1. Materials characterization of pristine YSZ/WOx interface. **a,** High resolution transmission electron microscope (HRTEM) image of YSZ/WOx interface. **b**, Selected area electron diffraction (SAED) pattern shows crystallinity of WOx. **c**, SAED pattern of YSZ.

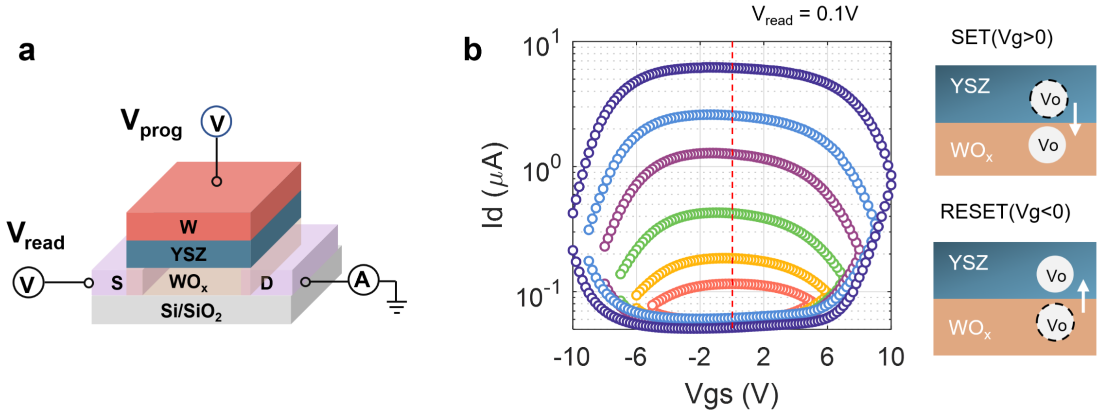

Supplementary Figure 2. Transfer characteristics of ECRAMs. **a**, Test set-up for transfer I-V curves, the gate electrode was scanned by voltages (V_{gs}) with a loop of 0-V_{max}-0-V_{min}-0, the channel current (I_d) was recorded by monitoring the drain electrode with constant read voltage while the source electrode was grounded. **b**, Transfer characteristics were measured within varying voltage ranges, indicating a non-volatile modulation of device conductance by using gate voltages. During set and reset process by polarized gate voltages, the oxygen vacancies are driven by electric field to dope and de-dope the channel oxides, resulting in channel conductance changes.

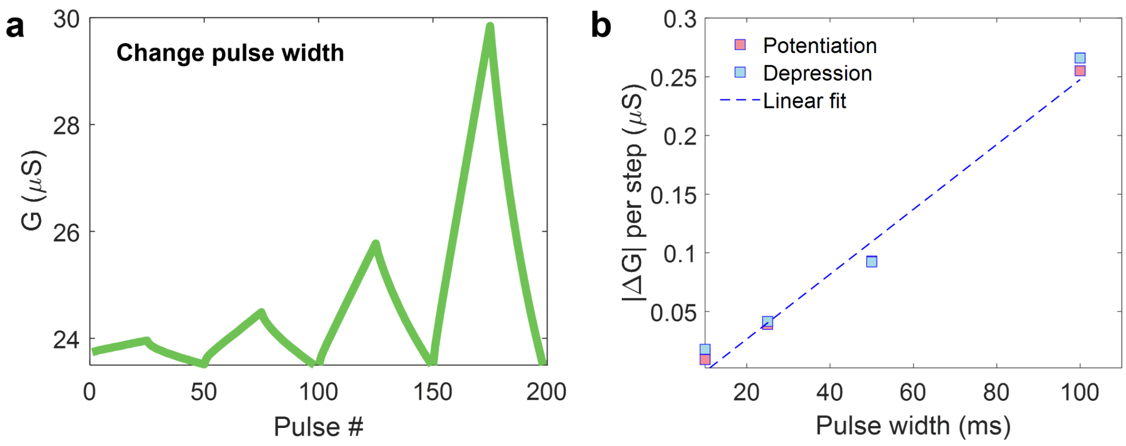

Supplementary Figure 3. Pulse width modulation. Linear and symmetric conductance update demonstrated in ECRAM with gate voltage pulses of varying pulse width (25 potentiation/depression pulses with an amplitude of ±5V, and width of 10, 25, 50, 100 ms). **a,** Conductance (G)-pulse number plot. **b,** Linear fit of calculated $|\Delta G|$ versus pulse width.

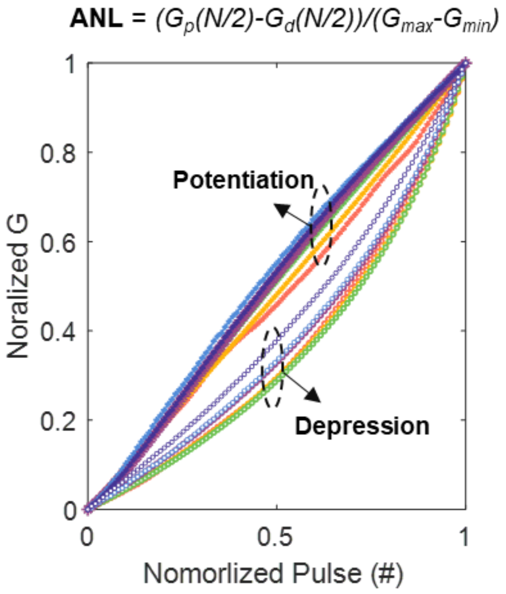

Supplementary Figure 4. Asymmetry-nonlinearity factor calculation. Normalized plot of pulse up/down cycles from different dynamic ranges. The asymmetry non-linearity factors in the six conductance windows are calculated as 0.12, 0.21, 0.25, 0.26, 0.23, and 0.20, respectively, ANL=($G_p(N/2)$-$G_d(N/2)$)/(G_{max}-G_{min}), demonstrating significantly better linearity over conventional non-volatile memory (NVM) devices such as ReRAM.

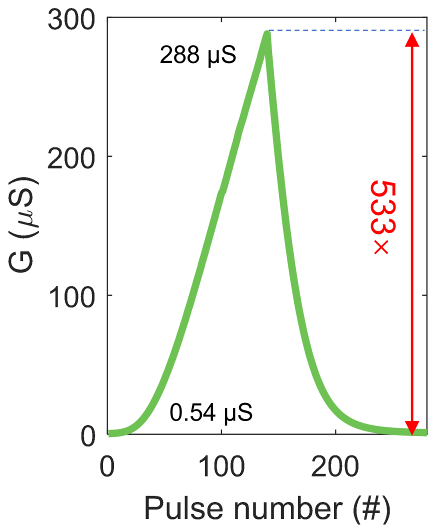

Supplementary Figure 5. Conductance modulation of an ECRAM over wide dynamic range. Programming ECRAM device with gate voltage pulses of 140 potentiation (+5 V/500 ms) and 140 depression (-5 V/500 ms), showing good symmetry and large dynamic range.

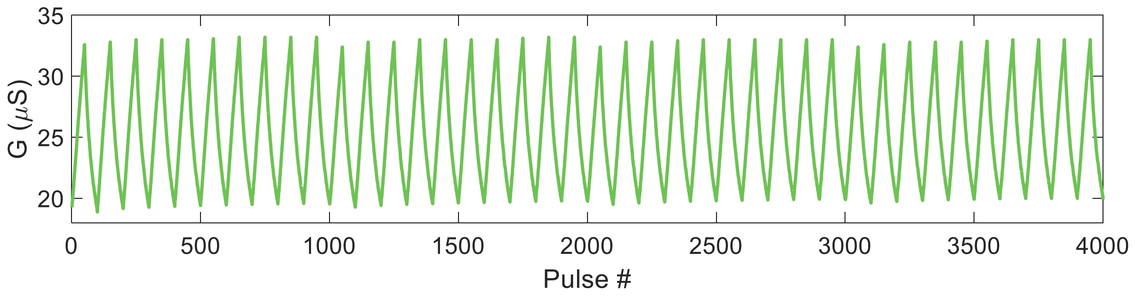

Supplementary Figure 6. Cycling performance of ECRAM device. In each cycle, the device was programmed with voltage pulses (50 potentiation/ 50 depression, ±5 V, 100 ms), and it was repeated consecutively for 40 cycles. The C2C variation σ/μ is calculated to be 0.8%.

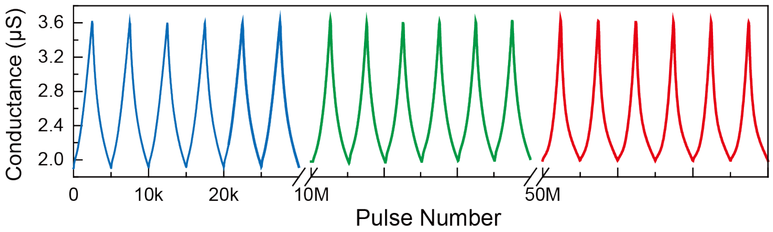

Supplementary Figure 7. Endurance test of the device. Pulse condition: $V_G = \pm 5V$, $t_{pulse} = 1ms$.

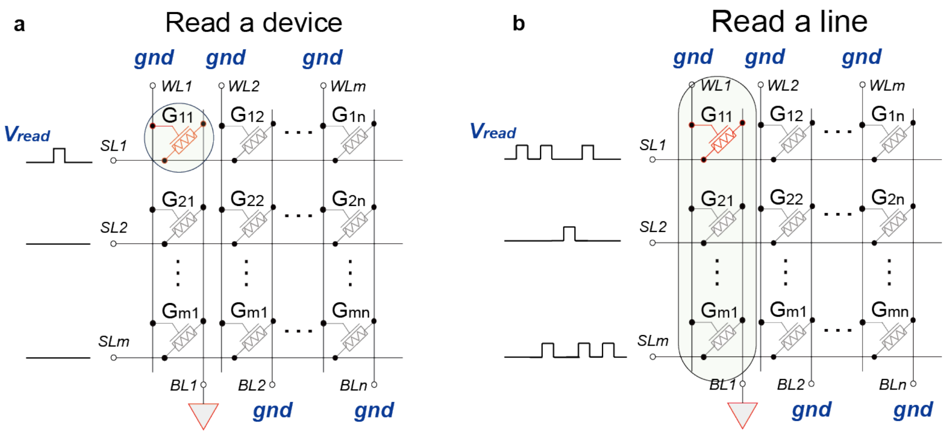

Supplementary Figure 8. Schematic diagrams of the read operations in an array. a, Schematic of read process of a single device. **b**, Schematic of read process of a whole line for VMM operations. During read operation, all word lines and unselected source/drain lines are grounded. Read pulses are applied to selected source lines, then record the output current along the selected drain line.

Write errors ($G_{actual} - G_{target}$, μS) during continuous pattern programming

a 1st pattern

	1	2	3	4	5	6	7	8	9	10
1	1.22	0.71	0.84	0.62	0.17	0.29	0.2	0.05	-0	-0.06
2	1.15	0.6	0.39	0.36	0.12	0.06	-0.07	-0.01	-0	-0.13
3	-0.4	-0.37	-0.19	-0.16	-0	-0.13	0.03	0.03	-0.13	-0.03
4	-0.4	-0.23	-0.19	-0.16	-0.12	-0.27	0.03	-0.01	0.05	-0.12
5	-0.13	-0.2	0.01	-0.07	0.03	-0.44	0.04	-0.06	-0.14	-0.14
6	0	-0.14	-0.28	-0.23	-0.24	-0.11	-0.37	-0.26	-0.26	-0.24
7	0.28	0.16	0.04	0.01	-0.02	-0.08	-0.2	-0.1	-0.05	-0.08
8	0.8	0.56	0.35	0.26	0.17	-0.13	0	0.05	0.2	-0.09
9	2.56	1.31	1.21	0.91	0.89	0.03	0.42	0.35	0.24	0.36
10	2.57	2.53	2.06	2.25	1.98	0.66	0.9	0.72	0.66	0.34

b 2nd pattern

	1	2	3	4	5	6	7	8	9	10
1	1.01	1.27	0.71	0.5	0.68	0.41	0.23	0.15	-0	0.04
2	0.57	0.74	0.56	0.52	0.4	0.34	0.24	-0.05	0.06	-0.04
3	0.19	0.23	0.15	-0.02	-0.13	0.09	-0.18	-0.22	-0.05	-0.17
4	0.06	-0.01	-0.09	-0.11	-0.09	-0.09	-0.13	-0.21	-0.04	-0.27
5	0.04	-0.02	-0.12	-0.15	-0.08	-0.02	-0.09	-0.1	-0.24	-0.16
6	0.15	0	-0.02	0	-0.07	-0.05	-0.13	-0.04	-0.13	-0.15
7	0.44	0.2	0.16	0.06	-0.04	-0.06	-0.1	0.01	-0.07	-0.08
8	0.04	0.16	-0.01	-0.09	-0.03	0.02	0	0.05	0.07	-0.03
9	1.48	1.92	1.26	0.35	0.2	0.41	0.12	-0.05	-0.05	-0.12
10	3.78	2.82	2.4	1.2	0.48	0.63	0.36	0.12	-0.01	-0.02

c 3rd pattern

	1	2	3	4	5	6	7	8	9	10
1	1.26	0.67	0.96	0.64	0.22	0.36	0.18	0.1	0.02	-0.03
2	1.15	0.63	0.53	0.41	0.14	0	-0.1	0.03	-0.06	-0.16
3	-0.44	-0.39	-0.21	-0.23	-0.14	-0.28	0.03	0	-0.18	-0.08
4	-0.35	-0.21	-0.24	-0.24	-0.2	-0.04	0.03	-0.01	-0.01	-0.08
5	-0.3	-0.31	-0.04	-0.06	-0.07	-0.32	0.01	-0.03	-0.08	-0.12
6	-0.01	-0.08	-0.18	-0.18	-0.13	-0.16	-0.2	-0.19	-0.17	-0.21
7	0.24	0.06	-1.11	0.05	-0.12	-0.08	-0.18	-0.11	-0.11	-0.12
8	1.03	0.47	0.23	-0.02	-0.05	-0.1	-0.08	-0.05	-0	-0.15
9	2.66	1.43	1.17	0.77	0.56	0.24	0.22	0.1	0.11	0.23
10	2.72	2.66	2.07	2.29	1.83	1.18	0.51	0.37	0.37	0.22

d 4th pattern

	1	2	3	4	5	6	7	8	9	10
1	0.89	1.16	0.69	0.42	0.69	0.24	0.2	0.14	-0.05	0
2	0.56	0.56	0.61	0.44	0.37	0.27	0.17	-0.02	0.01	-0.06
3	0.22	0.23	0.28	-0.02	-0.05	0.07	-0.09	-0.2	-0.01	-0.16
4	0.08	0.06	-0.06	-0.05	-0.12	-0.05	-0.14	-0.2	-0.06	-0.28
5	0.01	-0.01	-0.13	-0.18	-0.12	-0.02	-0.09	-0.11	-0.22	-0.16
6	-0	-0.05	0.01	-0.07	-0.06	-0.12	-0.17	-0.08	-0.12	-0.15
7	0.39	0.22	0.08	-0.05	-0.01	0.01	0	-0.08	-0.04	-0.03
8	1.16	0.09	-0.06	-0.17	-0.03	-0.04	-0.03	0.01	0.06	-0.04
9	1.37	1.87	1.09	0.37	0.21	0.38	0.2	0.01	-0	-0.08
10	3.7	2.78	2.2	1.17	0.48	0.63	0.41	0.1	-0.01	0.06

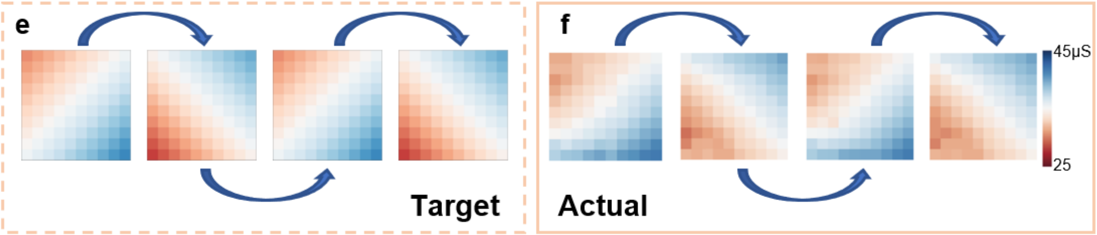

Supplementary Figure 9. Write errors (μS) during continuous pattern programming. write errors ($G_{actual} - G_{target}$, μS) derived from pattern writing of Fig. 3b in the main context, the write errors were mostly fell in 3% (for 90% of the devices) and 1μS. **a**, 1st pattern. **b**, 2nd pattern. **c**, 3rd pattern and **d**, 4th pattern. **e**, Illustration of pattern programming process, two patterns were continuously and repeatedly programmed into the ECRAM array from initial random states. **f**, Pattern programming results.

Write errors ($G_{actual} - G_{target}$, μS) during pattern programming with 1~3 cycles

a 1st cycle

	1	2	3	4	5	6	7	8	9	10
1	1.08	1.13	1.08	0.96	0.59	0.71	0.44	0.07	0.26	0.08
2	0.94	0.67	0.4	0.48	0.3	0.1	-0.01	-0.01	-0.04	-0.13
3	-0.53	-0.51	-0.36	-0.33	-0.4	-0.19	-0.03	-0.22	0.02	-0.04
4	-0.34	-0.24	-0.05	-0.17	-0.37	-0.07	-0.09	0.01	-0.33	-0.05
5	-0.18	-0.35	-0.09	-0.02	-0.32	-0.21	0.03	-0.04	-0.06	-0.11
6	-0.38	-0.24	-0.42	-0.3	-0.29	-0.02	-0.34	-0.25	-0.22	-0.35
7	0.63	0.11	0.05	-0.23	-0.14	-0.11	-0.31	-0.27	-0.21	-0.2
8	0.98	0.48	0.1	-0.08	-0.07	-0.13	-0.14	-0.21	-0.14	-0.19
9	2.62	1.44	1.57	0.91	0.73	0.42	0.19	-0.01	0.03	0.02
10	3.41	2.48	2.19	2.07	1.86	1.26	0.65	0.34	0.39	0.17

b 2nd cycle

	1	2	3	4	5	6	7	8	9	10
1	0.34	0.05	0.1	0.15	-0.05	0.1	-0.01	0.01	0.01	-0.06
2	0.24	0.05	0.01	0.09	0.03	-0	-0.06	-0.01	-0.06	-0.1
3	0.05	-0.02	-0.05	-0.12	-0.1	-0.15	-0.13	0.03	-0	0.01
4	-0.08	-0.11	-0.08	-0.09	-0.05	-0.08	0.01	-0.14	-0.07	-0.15
5	-0.13	-0.05	-0.29	-0.36	-0.05	-0.09	-0.08	0.01	-0.21	-0.16
6	-0.12	-0.2	-0.39	-0.49	-0.19	-0.12	-0.35	-0.24	-0.27	-0.29
7	0.43	0.04	0.26	-0.12	-0.14	-0.17	-0.23	-0.12	-0.17	-0.18
8	0.37	0.13	0.14	-0.02	-0.09	-0.05	-0.11	-0.09	-0.09	-0.18
9	-0.93	-0.03	0.28	0.46	0.56	0.26	0.06	0.07	0.06	-0.04
10	-2.04	0.39	0.81	0.6	0.74	0.88	0.82	0.55	0.35	0.31

c 3rd cycle

	1	2	3	4	5	6	7	8	9	10
1	0.13	0.11	0.08	-0.04	-0.06	0.05	-0.02	0.02	0.01	0.02
2	0.29	0.07	0.03	0.02	-0.02	-0.04	0.06	-0.01	-0.01	-0.05
3	0.15	-0.06	-0.01	0.08	-0.08	-0.06	0.01	-0.31	0.01	-0.07
4	-0.01	-0.19	-0.04	-0.06	-0.02	-0.05	-0.03	-0.07	-0.01	-0.11
5	-0.03	-0.07	-0.07	-0.09	-0.01	-0.11	-0.05	-0.22	-0.14	-0.11
6	-0.15	-0.15	-0.25	-0.29	-0.24	-0.09	-0.34	-0.2	-0.24	-0.26
7	-0.6	0.06	-0.58	0.14	0.01	0.03	-0.08	-0.03	-0.09	-0.12
8	-0.42	0.12	0.19	-0.06	-0.04	-0.05	-0.08	-0.06	-0.06	-0.12
9	-0.74	-0.59	0.01	0.32	0.12	0.22	0.06	0.05	-0.01	-0.01
10	-0	-1.78	-0.33	-0.66	0.52	0.52	0.54	0.56	0.23	0.11

d Programming results

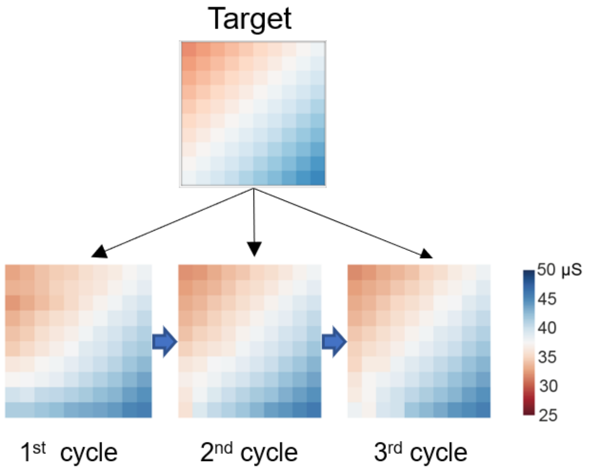

Supplementary Figure 10. Write errors of pattern programming with 1~3 cycles. Write errors (μS) derived from pattern writing of Fig. 3d in the main context. **a**, open loop. **b**, with once feedback. **c**, with twice feedback. **d,** Target pattern and programming results after 1~3 cycles. The average write errors were reduced from 1.22% to 0.53% in the 2nd cycle to 0.41% after 3rd cycle, and most of the program errors fell within 0.1μS.

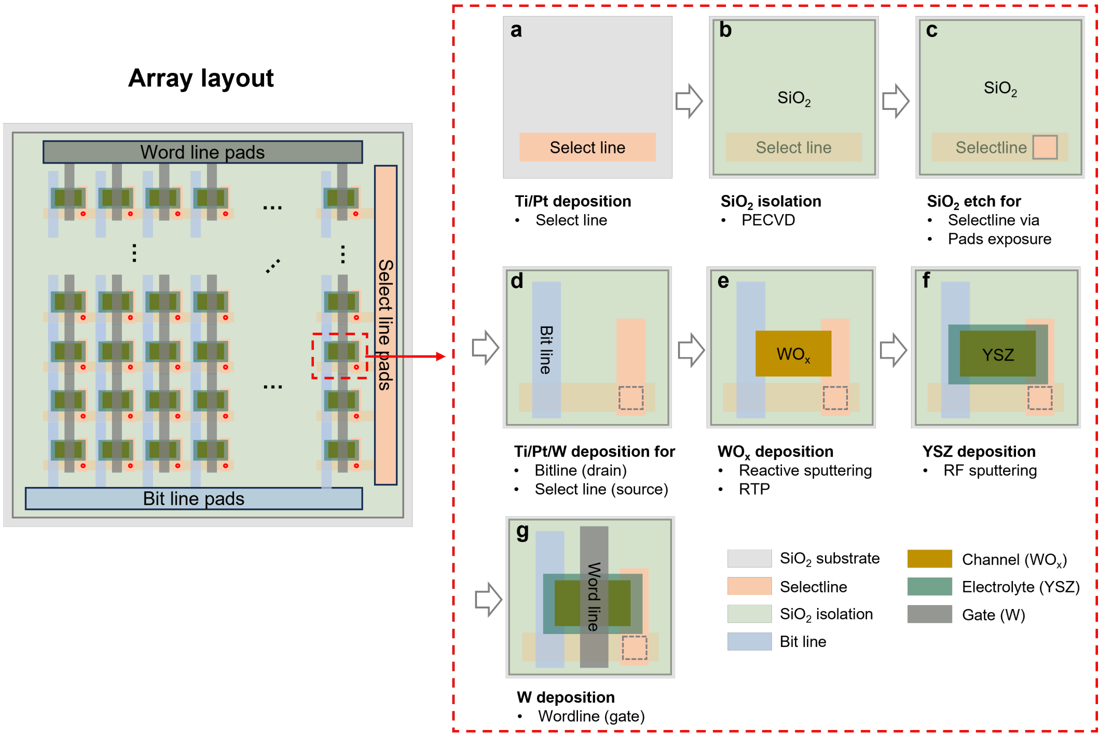

Supplementary Figure 11. Fabrication process of an ECRAM array. Left figure shows the layout of the ECRAM array, and the fabrication process of a unit cell is illustrated in right figure, including **a,** selectline: Ti (3 nm) / Pt (40 nm). **b**, SiO_2 isolation layer: PECVD SiO_2 (100 nm). **c**, SiO_2 dry etch for selectline via and testing pads exposure. **d**, Source contacts (connected through selectline vias) and bitlines: Ti (3 nm) / Pt (40 nm) / W (5 nm). **e**, WO_x (100 nm) channels: reactive sputtering from W target with an Ar/O_2 ratio of 4: 3, followed with rapid thermal process (RTP) at 400 °C for 30 seconds. **f**, YSZ (50 nm) electrolyte layers: RF sputtering with 8 wt. % yttria-stabilized-zirconia target. **g**, Word lines (gate): W (50 nm).

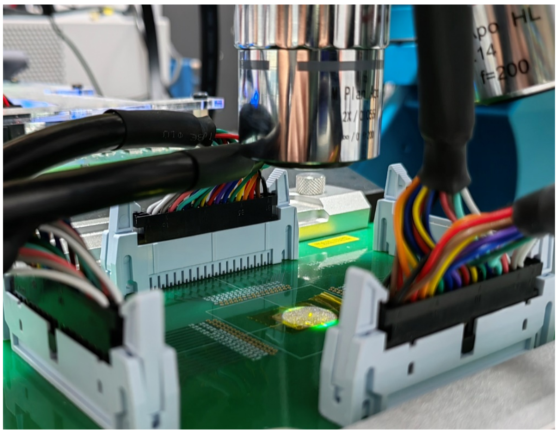

Supplementary Figure 12. Experimental set-up for array operation. The measured array samples were directly connected with a 128-pins probe card, then it could communicate with PCB testing system. Python based programs were customized to program/read the array samples, including basic write/read, vector-matrix multiplication, and extended array operations.

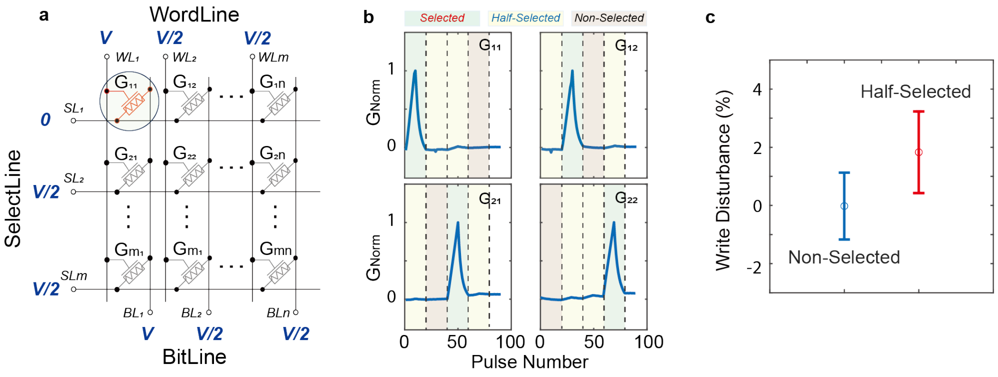

Supplementary Figure 13. Evaluation of crosstalk disturbance in half-selected ECRAM devices. a, Schematic of 1/2V programing scheme, 0V, V/2 and V were used to achieve half-selection. A more efficient scheme would be -1/2V, 0V and 1/2V but was not supported by the current system. **b**, Conductance update of four nearest devices in the array. **c,** Mean and standard deviation of relative conductance change in non-selected and half-selected devices compared to selected devices.

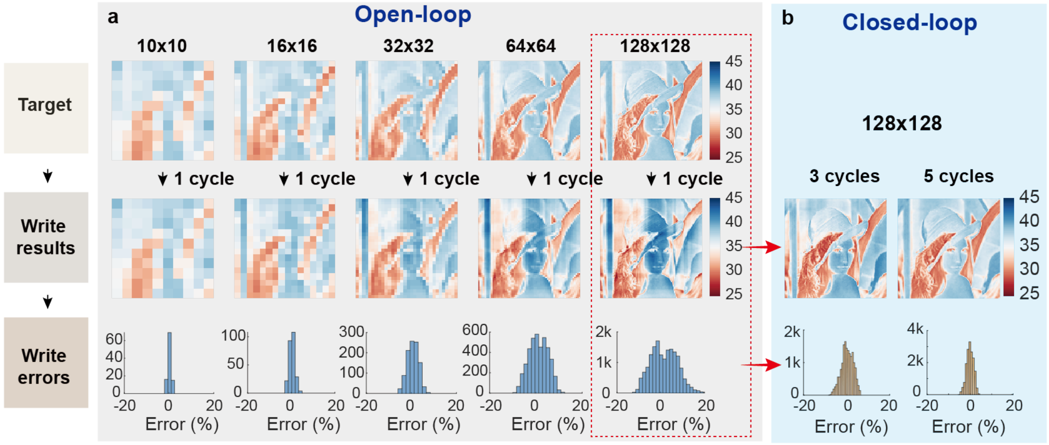

Supplementary Figure 14. Simulation of half-selected disturbance in larger arrays. **a,** Open-loop programming of down-sampled Lenna images into different sizes of ECRAM array. Half-selected devices were also updated in each programming cycle accounting for crosstalk disturbance. A half-voltage disturbance value of 3.0% was used to simulate the programming accuracy under slightly more challenging conditions than our experimental conditions. **b,** Iterative closed-loop programming in 128×128 array for 3 and 5 cycles, showing convergence of programming in larger arrays in the appearance of crosstalk interferences. For all simulations, the matrix started from randomly initialized values and each device was updated in series. The simulation code was programmed using MATLAB and intended as a coarse evaluation of the crosstalk effect. The simulation did not include the impact from line resistance and peripheral circuitry.

Supplementary Table 1. Summary of input parameters for in-situ training tasks.

#	Input parameters	Descriptions
1	Cap-shape	bell, conical, convex, flat, knobbed, sunken
2	Cap-surface	fibrous, grooves, scaly, smooth
3	Cap-color	brown, buff, cinnamon, gray, green, pink, purple, red, white, yellow
4	Bruises	bruises, no
5	Odor	almond, anise, creosote, fishy, foul, musty, none, pungent, spicy
6	Gill-attachment	attached, descending, free, notched
7	Gill-spacing	close, crowded, distant
8	Gill-size	Broad, narrow
9	Gill-color	black, brown, buff, chocolate, gray, green, orange, pink, purple, red, white, yellow
10	Stalk-shape	enlarging, tapering
